# Matrix Transformations between Certain Sequence Spaces over the Non-Newtonian Complex Field

**DOI:** 10.1155/2014/705818

**Published:** 2014-06-19

**Authors:** Uğur Kadak, Hakan Efe

**Affiliations:** ^1^Department of Mathematics, Faculty of Sciences, Gazi University, 06500 Ankara, Turkey; ^2^Department of Mathematics, Faculty of Sciences and Arts, Bozok University, 66100 Yozgat, Turkey

## Abstract

In some cases, the most general linear operator between two sequence spaces is given by an infinite matrix. So the theory of matrix transformations has always been of great interest in the study of sequence spaces. In the present paper, we introduce the matrix transformations in sequence spaces over the field ℂ^*^ and characterize some classes of infinite matrices with respect to the non-Newtonian calculus. Also we give the necessary and sufficient conditions on an infinite matrix transforming one of the classical sets over ℂ^*^ to another one. Furthermore, the concept for sequence-to-sequence and series-to-series methods of summability is given with some illustrated examples.

## 1. Introduction

The theory of sequence spaces is the fundamental of summability. Summability is a wide field of mathematics, mainly in analysis and functional analysis, and has many applications, for instance, in numerical analysis to speed up the rate of convergence, in operator theory, the theory of orthogonal series, and approximation theory. This subsection serves as a motivation of what follows. The classical summability theory deals with the generalization of the convergence of sequences or series of real or complex numbers. The idea is to assign a limit of some sort to divergent sequences or series by considering a transform of a sequence or series rather than the original sequence or series. One can ask why we employ the special transformations represented by infinite matrices instead of general linear operators. The answer to this question is that, in many cases, the most general linear operators between two sequence spaces are given by an infinite matrix. Many authors have extensively developed the theory of the matrix transformations between some sequence spaces we refer the reader to [1–13].

As an alternative to the classical calculus, Grossman and Katz [14–16] introduced the non-Newtonian calculus consisting of the branches of geometric, quadratic, and harmonic calculus, and so forth. All these calculi can be described simultaneously within the framework of a general theory. We decided to use the adjective* non-Newtonian* to indicate any of calculi other than the classical calculus. Every property in classical calculus has an analogue in non-Newtonian calculus which is a methodology that allows one to have a different look at problems which can be investigated via calculus. In some cases, for example, for wage-rate (in dollars, euro, etc.) related problems, the use of bigeometric calculus which is a kind of non-Newtonian calculus is advocated instead of a traditional Newtonian one.

Bashirov et al. [17, 18] have recently concentrated on the non-Newtonian calculus and gave the results with applications corresponding to the well-known properties of derivatives and integrals in the classical calculus. Also, Uzer [19] has extended the non-Newtonian calculus to the complex valued functions and was interested in the statements of some fundamental theorems and concepts of multiplicative complex calculus and demonstrated some analogies between the multiplicative complex calculus and classical calculus by theoretical and numerical examples. Further, Misirli and Gurefe have introduced multiplicative Adams Bashforth-Moulton methods for differential equations in [20]. Quite recently, Kadak [21, 22] have determinated Köthe-Toeplitz dual between classical sets of sequences over the non-Newtonian complex field and have constructed Hilbert spaces over the non-Newtonian field.

The main purpose of the present paper is to characterize some matrix classes between certain sequence spaces over the non-Newtonian complex field.

## 2. *β*-Arithmetics and Some Related Applications

A* generator* is a one-to-one function whose domain is *R* and whose range is a subset of *B*⊆*R*, the set of real numbers. Each generator generates exactly one arithmetic, and, conversely, each arithmetic is generated by exactly one generator. For example, the identity function generates classical arithmetic and exponential function generates geometric arithmetic. As a generator, we choose the function *β* such that its basic algebraic basic algebraic operations are defined as follows:

(1) β-addition  x +¨ y=β{β−1(x)+β−1(y)};β-subtraction  x −¨ y=β{β−1(x)−β−1(y)},β-multiplication  x ×¨ y=β{β−1(x)×β−1(y)};β-division  x /¨ y,xy∶=β{β−1(x)÷β−1(y)}(β−1(y)≠0),β-order  x <¨ y⟺β−1(x)<β−1(y),

for all *x*, *y* ∈ *R*_*β*_ where the non-Newtonian real field *R*_*β*_∶ = {*β*{*x*} : *x* ∈ *R*} as in [23].

The *β*-positive real numbers, denoted by *R*_*β*_^+^, are the numbers *x* in *R*_*β*_ such that $\ddot{0}\;\ddot{<}\;x$; the *β*-negative real numbers, denoted by *R*_*β*_^−^, are those for which $x\ddot{<}\ddot{0}$. The *beta*-zero, $\ddot{0}$, and the *beta*-one, $\ddot{1}$, turn out to be *β*(0) and *β*(1). Further, $\beta (-p)=\beta \{\beta^{-1}(\ddot{p})\}=\ddot{-}\ddot{p}$ holds for all *p* ∈ *Z* ^+^. Thus the set of all *β*-integers turns out to be the following:

(2) Zβ={…,β(−2),β(−1),β(0),β(1),β(2),…}={…,−¨2¨,−¨1¨,0¨,1¨,2¨,…}.

**Definition 1 (see [21]).**

Let *X* be a nonempty set and let d* : *X* × *X* → *R*_*β*_ be a function such that for all *x*, *y*, *z* ∈ *X*, then the following axioms hold:

(NM1) $d^{*}(x,y)=\ddot{0}$ if and only if *x* = *y*,
(NM2) d*(*x*, *y*) = d*(*y*, *x*),
(NM3) $d^{*}(x,y)\;\;\ddot{\leq }\;\;d^{*}(x,z)\;\;\ddot{+}\;\;d^{*}(z,y)$.

Then, the pair (*X*, d*) and d* are called a non-Newtonian metric space and a non-Newtonian metric on *X*, respectively.

**Definition 2 (see [23]).**

Let *X* = (*X*, d*) be a non-Newtonian metric space. Then the basic notions can be defined as follows.

- A sequence *x* = (*x*_*k*_) is a function from the set *N* into the set *R*_*β*_. The *β*-real number *x*_*k*_ denotes the value of the function at *k* ∈ *N* and is called the* k*th term of the sequence.
- A sequence (*x*_*n*_) in a metric space *X* = (*X*, d*) is said to be ∗-convergent if for every given $\varepsilon \;\ddot{>}\;\ddot{0}$ there exists an *n*_0_ = *n*_0_(*ε*) ∈ *N* and *x* ∈ *X* such that $d^{*}(x_{n},x)\;\;\ddot{<}\;\;\varepsilon$ for all *n* > *n*_0_ and is denoted by *lim⁡_*n*→*∞*_ *x*_*n*_ = *x* or $x_{n}\overset{*}{\to }x$, as *n* → *∞*.
- A sequence (*x*_*n*_) in *X* = (*X*, d*) is said to be non-Newtonian Cauchy (∗-Cauchy) if for every $\varepsilon \ddot{>}\ddot{0}$ there is an *n*_0_ = *n*_0_(*ε*) ∈ *N* such that $d^{*}(x_{n},x_{m})\;\;\ddot{<}\;\;\varepsilon$ for all *m*, *n* > *n*_0_.

**Remark 3 (see [21]).**

Let $\ddot{b}\in B\subseteq R$. Then the number $\ddot{b}\ddot{\times }\ddot{b}$ is called the *β*-square and is denoted by ${\ddot{b}}^{\ddot{2}}$. Let $\ddot{b}\in R_{\beta }^{+}$. Then $\beta \{\sqrt{\beta^{-1}(\ddot{b})}\}$ is called the *β*-square root of $\ddot{b}$ and is denoted by $\sqrt[{..}]{\ddot{b}}$. Further, for each $\ddot{b}\in B$ we can write ${\ddot{b}}^{\ddot{p}}=\beta \{{\left[\beta^{-1}(\ddot{b})\right]}^{p}\}=\beta \{b^{p}\}$ for all *p* ∈ *N*.

The *β*-absolute value of a number *x* in *B* ⊂ *R*(*N*) is defined as *β*(|*β* ^−1^(*x*)|) and is denoted by |*x*|_*β*_. For each number *x* in *A* ⊂ *R*(*N*), $\sqrt[{..}]{{\ddot{x}}^{\ddot{2}}}=|x{|}_{\beta }=\beta (|\beta^{-1}(x)|)$. Then we say

(3) $$\begin{array}{c} {\left|x\right|}_{\beta }=\{\begin{array}{cc} x, & x\ddot{>}\ddot{0}\\ \ddot{0,} & x=\ddot{0}=\beta \left\{\left|\beta^{-1}\left(x\right)\right|\right\}\\ \ddot{0}\;\ddot{-}\;x, & x\ddot{<}\ddot{0}. \end{array} \end{array}$$

The non-Newtonian distance between two numbers *x*_1_ and *x*_2_ is defined by $|x_{1}\;\ddot{-}\;x_{2}{|}_{\beta }=\beta \{|\beta^{-1}(x_{1})-\beta^{-1}(x_{2})|\}=\beta \{|\beta^{-1}(x_{2})-\beta^{-1}(x_{1})|\}=|x_{2}\ddot{-}x_{1}{|}_{\beta }$. Similarly, by taking into account the definition for *alpha*-generator in (3), one can conclude that the equality $|x_{1}\;\dot{-}\;x_{2}{|}_{\alpha }=|x_{2}\;\dot{-}\;x_{1}{|}_{\alpha }$ holds for all *x*_1_, *x*_2_ ∈ *R*.

Now, we give a new type calculus for non-Newtonian complex terms, denoted by ∗-calculus, which is a branch of non-Newtonian calculus. From now on we will use ∗-calculus type with respect to two arbitrarily selected generator functions.

### 2.1. ∗-Arithmetic

Suppose that *α* and *β* are two arbitrarily selected generators and (“star-”) also is the ordered pair of arithmetics (*β*-arithmetic, *α*-arithmetic). The sets $\left(B,\ddot{+},\ddot{-},\ddot{\times },\ddot{/}\right)$ and $\left(A,\dot{+},\dot{-},\dot{\times },\dot{/}\right)$ are complete ordered fields and *beta*(*alpha*)-generator generates *beta*(*alpha*)-arithmetics, respectively. Definitions given for *β*-arithmetic are also valid for *α*-arithmetic.

The important point to note here is that *α*-arithmetic is used for arguments and *β*-arithmetic is used for values; in particular, changes in arguments and values are measured by *α*-differences and *β*-differences, respectively. The operators of this calculus type are applied only to functions with arguments in *A* and values in *B*. The ∗-limit of a function *f* at an element *a* in *A* is, if it exists, the unique number *b* in *B* such that for every sequence (*a*_*n*_) of arguments of *f* distinct from *a*, if (*a*_*n*_) is *α*-convergent to *a*, then {*f*(*a*_*n*_)}  *β*-converges to *b* and is denoted by *lim⁡_*x*→*a*_ *f*(*x*) = *b*. That is,

(4) $$\begin{array}{c} {\;}^{*}\underset{x\to a}{\lim}f\left(x\right)=b⟺\forall \varepsilon \ddot{>}\ddot{0},\;\;\exists \delta \;\dot{>}\;\dot{0}∋{\left|f\left(x\right)\;\ddot{-}\;b\right|}_{\beta }\ddot{<}\varepsilon \\ \forall x\in A,\;{\left|x\dot{-}a\right|}_{\alpha }\dot{<}\delta . \end{array}$$

A function *f* is ∗-continuous at a point *a* in *A* if and only if *a* is an argument of *f* and *lim⁡_*x*→*a*_⁡*f*(*x*) = *f*(*a*). When *α* and *β* are the identity function *I*, the concepts of ∗-limit and ∗-continuity are identical with those of classical limit and classical continuity.

The isomorphism from *α*-arithmetic to *β*-arithmetic is the unique function *ι* (iota) that possesses the following three properties.

(i) *ι* is one to one.
(ii) *ι* is from *A* onto *B*.
(iii) For any numbers *u* and *v* in *A*,
  (5) $$\begin{array}{c} \iota \left(u\;\dot{+}\;v\right)=\iota \left(u\right)\;\ddot{+}\;\iota \left(v\right),\;\;\iota \left(u\;\dot{-}\;v\right)=\iota \left(u\right)\;\ddot{-}\;\iota \left(v\right),\\ \iota \left(u\;\;\dot{\times }\;\;v\right)=\iota \left(u\right)\ddot{\times }\iota \left(v\right),\;\;\iota \left(u\dot{/}v\right)=\iota \left(u\right)\ddot{/}\iota \left(v\right);\\ v\neq \dot{0},\;u\leq v⟺\iota \left(u\right)\;\;\ddot{\leq }\;\;\iota \left(v\right). \end{array}$$

It turns out that *ι*(*x*) = *β*{*α* ^−1^(*x*)} for every *x* in *A* and that $\iota (\dot{n})=\ddot{n}$ for every integer *n*. Since, for example, $u\;\;\dot{+}\;\;v=\iota^{-1}\{\iota (u)\;\;\ddot{+}\;\;\iota (v)\}$, it should be clear that any statement in *α*-arithmetic can readily be transformed into a statement in *β*-arithmetic.

### 2.2. Non-Newtonian Complex Field and Some Inequalities

Let $\alpha (a)=\dot{a}\in (A,\dot{+},\dot{-},\dot{\times },\dot{/})$ and $\beta (b)=\ddot{b}\in (B,\ddot{+},\ddot{-},\ddot{\times },\ddot{/})$ be arbitrarily chosen elements from corresponding arithmetics. Then the ordered pair $\left(\dot{a},\ddot{b}\right)$ is called a ∗-point. The set of all ∗-points is called the set of ∗-complex numbers and is denoted by C*; that is,

(6) $$\begin{array}{c} C^{*}∶=\left\{z^{*}=\left(\dot{a},\ddot{b}\right)\;|\;\dot{a}\in A\subseteq R,\ddot{b}\in B\subseteq R\right\}. \end{array}$$

Define the binary operations addition (⊕) and multiplication (⊙) of ∗-complex numbers $z_{1}^{*}=({\dot{a}}_{1},{\ddot{b}}_{1})$ and $z_{2}^{*}=({\dot{a}}_{2},{\ddot{b}}_{2})$ as follows:

(7) ⊕:  C∗×C∗⟶C∗(z1∗,z2∗)⟼z1∗⊕z2∗=(α{a1+a2},β{b1+b2})=(a˙1 +˙ a˙2,b¨1 +¨ b¨2)⊙: C∗×C∗⟶C∗(z1∗,z2∗)⟼z1∗⊙z2∗=(α{a1a2−b1b2},β{a1b2+b1a2}),

where ${\dot{a}}_{1},{\dot{a}}_{2}\in A$ and ${\ddot{b}}_{1},{\ddot{b}}_{2}\in B$.

**Theorem 4 (see [24]).**

(C*, ⊕, ⊙) is a field.

Following Grossman and Katz [15], we can give the definition of ∗-distance and some applications with respect to the ∗-calculus which is a kind of calculi of non-Newtonian calculus.

The ∗-distance d* between two arbitrarily elements $z_{1}^{*}=({\dot{a}}_{1},{\ddot{b}}_{1})$ and $z_{2}^{*}=({\dot{a}}_{2},{\ddot{b}}_{2})$ of the set C* is defined by

(8) d∗:  C∗×C∗⟶[0¨,∞)=B′⊂B(z1∗,z2∗)⟼d∗(z1∗,z2∗)=[ι(a˙1 −˙ a˙2)]2¨+¨(b¨1 −¨ b¨2)2¨..=β{(a1−a2)2+(b1−b2)2}.

Up to now, we know that C* is a field and the distance between two points in C* is computed by the function d*, defined by (8).

**Definition 5 (see [21]).**

Given a sequence (*z*_k_*) of ∗-complex numbers, the formal notation

(9) $$\begin{array}{c} {}_{{{{}_{}}_{}}_{*}}\overset{\infty }{\underset{k=0}{\sum }}z_{k}^{*}=z_{0}^{*}⊕z_{1}^{*}⊕z_{2}^{*}⊕\cdots ⊕z_{k}^{*}⊕\cdots ,\;\forall k\in N, \end{array}$$

is called an infinite series with ∗-complex terms, or simply complex *N*-series. Also, for integers *n* ∈ *N*, the finite ∗-sums *s*_n_* = _∗_∑_*k*=0_^*n*^ *z*_k_* are called the partial sums of complex *N*-series. If the sequence ∗-converges to a complex number s* then we say that the series  ∗-converges and write s* =  _∗_∑_*n*=0_^*∞*^ *z*_n_*. The number s* is then called the ∗-sum of this series. If (*s*_*n*_)  ∗-diverges, we say that the series ∗-diverges, or that it is ∗-divergent.

**Remark 6 .**

Given a sequence (*x*_*k*_) of *β*-real numbers *R*_*β*_, the formal notation

(10) $$\begin{array}{c} {\;}_{{}_{{}_{{}_{\beta }}}}\overset{\infty }{\underset{k=0}{\sum }}x_{k}=\beta \left\{\overset{\infty }{\underset{k=0}{\sum }}\beta^{-1}\left\{x_{k}\right\}\right\}=x_{1}\;\ddot{+}\;x_{2}\;\ddot{+}\;x_{3}\;\ddot{+}\cdots \ddot{+}\;x_{k}\;\ddot{+}\cdots \end{array}$$

is called an infinite non-Newtonian series with *β* real terms. Also, for integers *n* ∈ *N*, the finite sums *s*_*n*_= _*β*_∑_*k*=0_^*n*^ *x*_*k*_ are called the partial sums of the *N*-series. If the sequence *β*-converges to a real number *s* then we say that the series *β*-converges and write *s*=_*β*_∑_*n*=0_^*∞*^ *x*_*k*_. The number *s* is then called the sum of this series. If (*s*_*n*_)  *β*-diverges, we say that the *N*-series is *β*-divergent.

**Proposition 7 (see [24]).**

For any *z*_1_*, *z*_2_* ∈ C*. Then the following statements hold.

(i) $\ddot{||}z_{1}^{*}⊕z_{2}^{*}\ddot{||}\;\;\ddot{\leq }\;\;\ddot{||}z_{1}^{*}\ddot{||}\;\ddot{+}\;\ddot{||}z_{2}^{*}\ddot{||}$. (∗*-triangle inequality)*
(ii) $\ddot{||}z_{1}^{*}⊙z_{2}^{*}\ddot{||}\;=\;\ddot{||}z_{1}^{*}\ddot{||}\;\ddot{\times }\;\ddot{||}z_{2}^{*}\ddot{||}$.
(iii) *Let* $p\;\ddot{>}\;\ddot{1}$ *and z*_k_*, *t*_k_* ∈ C* for k ∈ {0,1, 2,3,…, *n*}*. Then,*
  (11) (∗∑k=0n||¨zk∗⊕tk∗||¨p)1/¨p ≤¨(∗∑k=0n||¨zk∗||¨p‍)1/¨p+¨(∗∑k=0n||¨tk∗||¨p)1/¨p(Minkowski's  inequality).

Folllowing Tekin and Başar [24], we can give the ∗-norm and next derive some required inequalities in the sense of non-Newtonian complex calculus.

Let z* ∈ C* be an arbitrary element. The distance function d*(z*, θ*) is called ∗-norm of z* and is denoted by $\ddot{||}\cdot \ddot{||}$. In other words,

(12) ||¨z∗||¨=d∗(z∗,θ∗)=[ι(a˙ −˙ 0˙)]2¨ +¨ (b¨ −¨ 0¨)2¨..=β{a2+b2},

where $z^{*}=(\dot{a},\ddot{b})$ and $\theta^{*}=(\dot{0},\ddot{0})$. Moreover, since for all *z*_1_*, *z*_2_* ∈ C* we have $d^{*}(z_{1}^{*},z_{2}^{*})=\ddot{||}z_{1}^{*}⊖z_{2}^{*}\ddot{||}$ which d* is the induced metric from $\ddot{||}\cdot \ddot{||}$ norm.

**Definition 8 (see [21], complex conjugate).**

Let $z^{*}=(\dot{a},\ddot{b})\in C^{*}$. We define the ∗-complex conjugate ${\bar{z}}^{*}$ of z* by ${\bar{z}}^{*}=(\alpha \{a\},\beta \{-\beta^{-1}(\ddot{b})\})=(\dot{a},\ddot{-}\ddot{b})$. Conjugation changes the sign of the imaginary part of z* but leaves the real part the same. Thus,

(13) Re(z¯∗)=Re(z∗)=(z∗⊕z¯∗)/˙2˙=a˙,Im(z¯∗)=−¨Im(z∗)=(z∗⊖z¯∗)/¨2¨=b¨.

**Theorem 9 (see [24]).**

(C*, d*) is a complete metric space, where d* is defined by (8).

**Corollary 10 (see [24]).**

C* is a Banach space with the ∗-norm $\ddot{||}\cdot \ddot{||}$ defined by $\ddot{||}z^{*}\ddot{||}=\sqrt[{..}]{{\left[\iota (\dot{a})\right]}^{\ddot{2}}+{\left(\ddot{b}\right)}^{\ddot{2}}}$; $z^{*}=(\dot{a},\ddot{b})\in C^{*}$.

## 3. Non-Newtonian Infinite Matrices

A non-Newtonian infinite matrix *A* = (*a*_ij_*) of non-Newtonian complex numbers is a double sequence of complex numbers defined by a function *A* from the set *N* × *N* into the complex field C*, where *N* denotes the set of natural numbers, that is, *N* = {0,1, 2,…}. The complex number *a*_ij_* denotes the value of the function at (*i*, *j*) ∈ *N* × *N* and is called the entry of the matrix in the* i*th row and* j*th column.

The addition (⊕) and scalar multiplication (⊙) of the infinite matrices $A=(a_{ij}^{*})=({\dot{\epsilon }}_{ij},{\ddot{\delta }}_{ij})$ and $B=(b_{ij}^{*})=({\dot{\mu }}_{ij},{\ddot{\eta }}_{ij})$ are defined by

(14) A⊕B=(aij∗⊕bij∗)=(ϵ˙ij,δ¨ij)⊕(μ˙ij,η¨ij)=(ϵ˙ij +˙ μ˙ij,δ¨ij +¨ η¨ij)=(α{ϵij+μij},β{δij+ηij}),λ∗⊙A=(λ∗⊙aij∗)=(λ˙,λ¨)⊙(ϵ˙ij,δ¨ij)=(α{λϵij−λδij},β{λϵij+λδij}),

where the elements *ε*, *δ*, *μ*, *η* are in *R* and $\lambda^{*}=(\dot{\lambda },\ddot{\lambda })$ is a non-Newtonian scalar in C*. The product *A*⊙*B* of the infinite matrices *A* = (*a*_ij_*) and *B* = (*b*_ij_*) is defined by

(15) (A⊙B)ij=∗∑k=0∞aik∗⊙bkj∗=(α{∑k=0∞(εikμkj−δikηkj)},β{∑k=0∞(εikηkj+δikμkj)}), ∀i,j∈N,

provided that the series on the right hand side of (15) ∗-converge for all *i*, *j* ∈ *N*, where (*A*⊙*B*)_*ij*_ denotes the entry of the matrix *A*⊙*B* in the* i*th row and* j*th column. For simplicity in notation, here and in what follows, the summation without limits runs from 0 to *∞*. On the other hand, the series on the right hand side of (15) ∗-converges if and only if

(16) $$\begin{array}{c} \underset{k}{\sum }\left(\varepsilon_{ik}\mu_{kj}-\delta_{ik}\eta_{kj}\right),\;\;\underset{k}{\sum }\left(\varepsilon_{ik}\eta_{kj}+\delta_{ik}\mu_{kj}\right) \end{array}$$

are convergent classically for all *k*, *n* ∈ *N*. However the series (15) may ∗-diverge for some, or all, values of *i*, *j*; the product *A*⊙*B* of the infinite matrices may not exist.

**Definition 11 (see [25]).**

Consider the following system of an infinite number of linear equations in infinitely many unknown *x*_0_*, *x*_1_*, *x*_2_*,… elements by _∗_∑_*k*_ *a*_ik_*⊙*x*_k_* = *y*_*i*_ for all *i* ∈ *N*. If we construct a non-Newtonian infinite matrix *A* = (*a*_ik_*) with the coefficients *a*_ik_* of the unknowns *x*_k_* and denote the ∗-vectors of unknowns and constants by *X* and *Y*, then the above sum can be expressed in matrix form as *A*⊙*X* = *Y*. Also *I*_∗_⊙*A* = *A*⊙*I*_∗_ = *A*, where *I*_∗_ = (*δ*_ij_*) is called ∗-unit matrix and is defined by

(17) $$\begin{array}{c} \delta_{ij}^{*}=\{\begin{array}{cc} 1^{*}=\left(\dot{1},\ddot{1}\right), & i=j,\\ \theta^{*}=\left(\dot{0},\ddot{0}\right), & i\neq j. \end{array} \end{array}$$

A very important application of infinite matrices is used in the theory of summability of divergent sequences and series which is considered based on non-Newtonian mean in next chapter. A simple example of the non-Newtonian Cesaro mean, denoted by ∗-Cesaro mean of order one, which is the analog of the well-known method of summability given below.

**Example 12 (Cesàro mean).**

Define the matrix *C*_1_* = (*c*_nk_*) by

(18) $$\begin{array}{c} c_{nk}^{*}=\{\begin{array}{cc} {\left(\frac{1}{n+1}\right)}^{*}, & 0\leq k\leq n,\\ \theta^{*}, & k>n. \end{array} \end{array}$$

If we choose the generator functions as *α* = exp⁡ and *β* = exp⁡ the calculus is bigeometric calculus [14, 15], then we obtain an infinite matrix with complex terms as follows:

(19) $$\begin{array}{c} \left(\begin{array}{cccccc} \left(e,e\right) & \left(1,1\right) & \cdots & \cdots & \cdots & \cdots \\ \left(\sqrt{e},\sqrt{e}\right) & \left(\sqrt{e},\sqrt{e}\right) & \left(1,1\right) & \cdots & \cdots & \cdots \\ \left(\sqrt[{3}]{e},\sqrt[{3}]{e}\right) & \left(\sqrt[{3}]{e},\sqrt[{3}]{e}\right) & \left(\sqrt[{3}]{e},\sqrt[{3}]{e}\right) & \left(1,1\right) & \cdots & \cdots \\ \vdots & \vdots & \vdots & \ddots & \vdots & \vdots \\ \left(\sqrt[{n+1}]{e},\sqrt[{n+1}]{e}\right) & \left(\sqrt[{n+1}]{e},\sqrt[{n+1}]{e}\right) & \left(\sqrt[{n+1}]{e},\sqrt[{n+1}]{e}\right) & \cdots & \left(\sqrt[{n+1}]{e},\sqrt[{n+1}]{e}\right) & \cdots \\ \vdots & \vdots & \vdots & \cdots & \vdots & \ddots \end{array}\right), \end{array}$$

where θ* = (*α*(0), *β*(0)) = (1,1) and *e* is a logarithmic number. The important point to note here is that the infinite matrix can be obtained in a similar way by using different generator functions above mentioned.

The ∗-zero matrix θ* is the matrix whose entries are all equal to θ*. Thus, it is obvious that *A*⊙θ* = θ*⊙*A* = θ*. But, as classical, *A*⊙*B* = θ* does not imply *A* = θ* or *B* = θ*. Further, the conjugate $\bar{A}$ of a complex matrix *A* = (*a*_ij_*) is the matrix $\bar{A}=({\bar{a}}_{ij}^{*})$ where ${\bar{a}}_{ij}^{*}$ is the conjugate of the complex number *a*_ij_* in Definition 8.

### 3.1. Non-Newtonian Matrix Transformations

Let *μ*_1_*, *μ*_2_* ⊂ w* and $A=(a_{nk}^{*})=({\dot{\varepsilon }}_{nk},{\ddot{\delta }}_{nk})$ be an infinite matrix of non-Newtonian complex numbers for all ${\dot{\varepsilon }}_{k}\in R_{\alpha }$ and ${\ddot{\delta }}_{k}\in R_{\beta }$. Then, we say that *A* defines a matrix mapping from *μ*_1_* into *μ*_2_* and denote it by writing *A* : *μ*_1_* → *μ*_2_*, if for every sequence *z* = (*z*_k_*) ∈ *μ*_1_* the sequence *A*⊙*z* = {(*Az*)_*n*_}, the *A*-transform of *z*, exists and is in *μ*_2_*, where

(20) A⊙z=(a00∗a01∗⋯a0k∗⋯a10∗a11∗⋯a1k∗⋯⋮⋮⋮⋯⋮⋯an0∗an1∗⋯ank∗⋯⋮⋮⋮⋯⋮⋯)⊙(z0∗z1∗⋮zk∗⋮)=(a00∗⊙z0∗⊗a01∗⊙z1∗⊕⋯a10∗⊙z0∗⊗a11∗⊙z1∗⊕⋯⋮an0∗⊙z0∗⊗an1∗⊙z1∗⊕⋯⋮)=(        ∗∑ka0k∗⊙zk∗        ∗∑ka1k∗⊙zk∗⋮        ∗∑kank∗⊙zk∗⋮)=((Az)0(Az)1⋮(Az)n⋮),

and, in this way, we transform the sequence $z=(z_{k}^{*})=({\dot{\mu }}_{k},{\ddot{\eta }}_{k})$, with ${\dot{\mu }}_{k}\in R_{\alpha }$ and ${\ddot{\eta }}_{k}\in R_{\beta }$, into the sequence {(*Az*)_*n*_} by

(21) (Az)n∶=      ∗∑kank∗⊙zk∗=(α{∑k(εnkμk−δnkηk)},β{∑k(εnkηk+δnkμk)‍})

for all *n* ∈ *N*_0_ and *ε*, *δ*, *μ*, *η* ∈ *R*. Thus, *A* ∈ (*μ*_1_* : *μ*_2_*) if and only if the series on the right side of (21) ∗-converges for each *n* ∈ *N* and every *z* ∈ *μ*_1_*, and we have *A*⊙*z* = {(*Az*)_*n*_}_*n*∈*N*_ ∈ *μ*_2_* for all *z* ∈ *μ*_1_*. On the other hand, we say *A* ∈ (*μ*_1_* : *μ*_2_*) if and only if the series ∑_*k*_(*ε*_*nk*_ *μ*_*k*_ − *δ*_*nk*_ *η*_*k*_) and ∑_*k*_(*ε*_*nk*_ *η*_*k*_ + *δ*_*nk*_ *μ*_*k*_) are convergent classically for all *k*, *n* ∈ *N*_0_. A sequence *z* is said to be *A*-summable to *γ* if *A*⊙*z*  ∗-converges to *γ* ∈ C* which is called as the *A*-*lim⁡ of *z*. We denote the* n*th row of a matrix *A* = (*a*_nk_*) by *A*_n_* for all *n* ∈ *N*; that is, *A*_n_*∶ = {*a*_nk_*}_*k*=0_^*∞*^ for all *n* ∈ *N*. Following Başar [25], we give some lines about ordinary and absolute summability of non-Newtonian complex numbers.

Let *A* = (*a*_nk_*) be an infinite matrix of non-Newtonian complex numbers throughout. We define two kinds of summability: ordinary and absolute summability, as shortly mentioned, below.

*(a) Ordinary Summability.* A sequence *z* = (*z*_*k*_) ∈ w* is said to be summable *A* to a *γ* ∈ C* if the *A*-*lim⁡ of *z* is $\gamma =({\dot{\gamma }}_{1},{\ddot{\gamma }}_{2})$ for all *γ*_1_, *γ*_2_ ∈ *R*; that is, *lim⁡_*n*→*∞*_ d*((*Az*)_*n*_, *γ*) = θ* which implies that

(22) $$\begin{array}{c} \underset{k}{\sum }\left(\varepsilon_{nk}\mu_{k}-\delta_{nk}\eta_{k}\right)⟶\gamma_{1},\\ \underset{k}{\sum }\left(\varepsilon_{nk}\eta_{k}+\delta_{nk}\mu_{k}\right)⟶\gamma_{2} \end{array}$$

in classical mean for each *k*, *n* ∈ *N*_0_. The matrix *A* defines a summability method *A* or a matrix transformation by (21).

*(b) Absolute Summability.* A sequence *z* = (*z*_*k*_) ∈ w* is said to be absolutely summable with index *p* to a number *ζ* ∈ C* if the series on the right hand side of (21) ∗-converge for each *n* ∈ *N* and

(23) $$\begin{array}{c} \overset{\infty }{\underset{n=0}{\sum }}d^{*}{\left({\left(Az\right)}_{n},\theta^{*}\right)}^{\ddot{p}}=\zeta \;\left(1<p<\infty \right). \end{array}$$

The Cesàro transform of a sequence *z* = (*z*_k_*) ∈ w* is given by *C*_1_*⊙*z* = {(*C*_1_*z)_*n*_}_*n*∈*N*_, where the Cesàro method *C*_1_* of one order is given by Example 12. Now, following Example 12, we may state the Cesàro summability with respect to the non-Newtonian calculus which is analogous to the classical Cesàro summable.

**Example 13 .**

Suppose that *z* = (*z*_k_*) is an infinite sequence defined by

(24) $$\begin{array}{c} z_{k}^{*}=\{\begin{array}{cc} 1^{*}, & k\;\;\text{even},\\ {⊖1}^{*}, & k\;\;\text{odd}, \end{array} \end{array}$$

where $⊖1^{*}=(\dot{-}\dot{1},\ddot{-}\ddot{1})\in C^{*}$. One can easily conclude that *z*_k_* ∈ *l*_∞_*∖c*. Then, since $\theta^{*}\ddot{\leq }\;\ddot{||}{\left(C_{1}^{*}z\right)}_{n}\ddot{||}\;\ddot{\leq }\;\;{\left(1/\left(n+1\right)\right)}^{*}$ for all *n* ∈ *N*, *lim⁡_*n*→*∞*_⁡(*C*_1_*z)_*n*_ = θ*. This means that the ∗-divergent sequence (*z*_k_*) is *C*_1_*-summable to θ*.

Tekin and Başar [24] have introduced the sets *l*_∞_*, c*, *c*_0_* and *l*_p_* of all bounded, convergent, null, and absolutely *p*-summable sequences over the complex field C* which correspond to the sets *l*_*∞*_, *c*, *c*_0_ and *l*_*p*_ over the complex field *C*, respectively. That is to say that

(25) l∞∗={z∗=(zk∗)∈ω∗:sup⁡k∈N||¨zk∗||¨<∞},c∗={z∗=(zk∗)∈ω∗:∃l∈C∗∋ ∗lim⁡k→∞zk∗=l},c0∗={z∗=(zk∗)∈ω∗: ∗lim⁡k→∞zk∗=θ∗},lp∗={z∗=(zk∗)∈ω∗:∗∑k=1∞||¨zk∗||¨p¨<∞}, (1≤p<∞).

It is not hard to show that the sets *l*_∞_*, c*, *c*_0_*, and *l*_p_* are the subspaces of the space ω*. This means that *l*_∞_*, c*, *c*_0_*, and *l*_p_* are classical sequence spaces over the field C* and complete metric spaces with corresponding metrics.

Quite recently, Kadak [21] have introduced the sets bs*, cs*, and *cs*_0_* consisting of the sets of all bounded, convergent, and null series based on the non-Newtonian calculus, as follows:

(26) bs∗∶={x=(xk)∈ω∗:||x||bs∗=sup⁡n∈N||¨∗∑k=0n‍xk||¨<∞},cs∗∶={x=(xk)∈ω∗:(      ∗∑k=0n‍xk)∈c∗},cs0∗∶={x=(xk)∈ω∗:(∗∑k=0n‍xk)∈c0∗},ω∗={x=(xk):xk∈C∗ ∀k∈N}.

**Theorem 14 (see [24]).**

The following statements hold.

(a) The sets *l*_∞_*, c*, *c*_0_*, and *l*_p_*; *p* ≥ 1 are sequence spaces.
(b) Let λ* denote any of the spaces *l*_∞_*, c*, and *c*_0_* and *z* = (*z*_k_*), *t* = (*t*_k_*) ∈ λ*. Define *d*_∞_* on the space λ* by $d_{\infty }^{*}(z,t)=\sup_{k\in N}\ddot{||}z_{k}^{*}⊖t_{k}^{*}\ddot{||}$. Then, (λ*, *d*_∞_*) is a complete metric space.
(c) The spaces *l*_∞_*, c*, and *c*_0_* are Banach spaces with the norm ||*z*||_∞_* defined by
  (27) $$\begin{array}{c} {||z||}_{\infty }^{*}∶=\underset{k\in N}{\sup}\ddot{||}z_{k}^{*}\ddot{||};\;z=\left(z_{k}^{*}\right)\in \lambda^{*},\\ \lambda \in \left\{l_{\infty },c,c_{0}\right\}. \end{array}$$
(d) The space *l*_p_* is Banach spaces with the norm ||*z*||_p_* defined by
  (28) $$\begin{array}{c} {||z||}_{p}^{*}∶={\left({}_{{{{}_{}}_{}}_{*}}\overset{\infty }{\underset{k=0}{\sum }}\ddot{||}z_{k}^{*}{\ddot{||}}^{\ddot{p}}\right)}^{\ddot{1}\ddot{/}\ddot{p}};\;z=\left(z_{k}^{*}\right)\in l_{p}^{*}. \end{array}$$

**Theorem 15 (see [21]).**

Let μ* denote any of the spaces bs*, cs*, and *cs*_0_*, and *z* = (*z*_k_*), *t* = (*t*_k_*) ∈ μ*. Define *d*_*∞*_^*N*^ on the space μ* by

(29) d∞N:μ∗×μ∗⟶Rβ(z,t)⟶d∞N(z,t)∶=sup⁡n∈N{d∗(      ∗∑k=0nzk∗,      ∗∑k=0ntk∗)}

for arbitrarily chosen *α*, *β* operators and corresponding function *ι* = *βα* ^−1^. Then, (μ*, *d*_*∞*_^*N*^) is a complete metric space.

**Corollary 16 (see [21]).**

The spaces bs*, cs*, and *cs*_0_* are Banach spaces with the norm ||*x*||_bs_* defined by

(30) $$\begin{array}{c} {||x||}_{bs}^{*}={||x||}_{cs}^{*}=\underset{n\in N}{\sup}\ddot{||}{}_{{{{\;}_{\;}}_{\;}}_{*}}\overset{n}{\underset{k=1}{\sum }}x_{k}\ddot{||};\\ x=\left(x_{n}\right)\in \lambda^{*},\;\lambda \in \left\{bs,cs,cs_{0}\right\}. \end{array}$$

**Theorem 17 (see [21]).**

Let *d*_Δ_ be defined on the space bv* by

(31) dΔ:bv∗×bv∗⟶Rβ(z,t)⟶dΔ(z,t)∶=      ∗∑k=0∞{d∗[(Δz)k′,(Δt)k′]},

where *z* = (*z*_*k*_), *t* = (*t*_*k*_) ∈ bv*, and (Δ*z*)_*k*_′ = *z*_*k*_ ⊖ *z*_*k*+1_. Then, (bv*, *d*_Δ_) is a complete metric space.

Firstly, we give the alpha-, beta-, and gamma-duals of a set λ* ⊂ ω* which are, respectively, denoted by {λ*}^*α*^, {λ*}^*β*^, and {λ*}^*γ*^, as follows:

(32) {λ∗}α∶={w=(wk∗)∈ω∗:w⊙z=(wk∗⊙zk∗)∈l1∗  ∀z=(zk∗)∈λ∗},{λ∗}β∶={w=(wk∗)∈ω∗:w⊙z=(wk∗⊙zk∗)∈cs∗  ∀z=(zk∗)∈λ∗},{λ∗}γ∶={w=(wk∗)∈ω∗:w⊙z=(wk∗⊙zk∗)∈bs∗  ∀z=(zk∗)∈λ∗},

where (*w*_k_*⊙*z*_k_*) is the coordinatewise product of ∗-complex numbers *w* and *z* for all *k* ∈ *N*. Then {λ*}^*β*^ is called beta-dual of λ* or the set of all convergence factor sequences of λ* in cs*. Firstly, we give a remark concerning with the ∗-convergence factor sequences.

**Theorem 18 (see [21]).**

The following statements hold.

- {*c*_0_*}^*β*^ = {c*}^*β*^ = {*l*_∞_*}^*β*^ = *l*_1_*.
- {*l*_1_*}^*β*^ = *l*_∞_*.

**Theorem 19 (see [21]).**

The following statements hold.

- {cs*}^*α*^ = {bv*}^*α*^ = {*bv*_0_*}^*α*^ = *l*_1_*.
- {cs*}^*β*^ = bv*, {bv*}^*β*^ = cs*, {*bv*_0_*}^*β*^ = bs*, {bs*}^*β*^ = *bv*_0_*.
- {cs*}^*γ*^ = bv*, {bv*}^*γ*^ = bs*, {*bv*_0_*}^*γ*^ = bs*, {bs*}^*γ*^ = bv*.

Now, we give the characterizations of some matrix classes and state the necessary and sufficient condition on non-Newtonian matrix transformations by using the results given on Köthe-Toeplitz duals in [21].

**Theorem 20 .**

The following statements hold:

(i) *A* = (*a*_*nk*_)∈(*l*_∞_* : *l*_∞_*) if and only if
  (33) $$\begin{array}{c} M=\underset{n\in N}{\sup}{}_{{{{\;}_{\;}}_{\;}}_{\beta }}\overset{}{\underset{k}{\sum }}\ddot{||}a_{nk}^{*}\ddot{||}\ddot{<}\infty . \end{array}$$
(ii) *A* = (*a*_*nk*_)∈(c* : *l*_∞_*) if and only if (33) holds.
(iii) *A* = (*a*_*nk*_)∈(*c*_0_* : *l*_∞_*) if and only if (33) holds.
(iv) *A* = (*a*_*nk*_)∈(*l*_p_* : *l*_∞_*) if and only if
  (34) $$\begin{array}{c} C=\underset{n\in N}{\sup}{}_{{{{\;}_{\;}}_{\;}}_{\beta }}\overset{}{\underset{k}{\sum }}{\ddot{||}a_{nk}^{*}\ddot{||}}^{\ddot{p}}\ddot{<}\infty ,\;\left(\ddot{0}\leq \ddot{p}<\infty \right). \end{array}$$

**Proof**

Since the proof can also be obtained in the similar way for other cases, to avoid the repetition of the similar statements, we prove only case (i).

Suppose that condition (33) holds and *x* = (*x*_*k*_) ∈ *l*_∞_*. In this situation, since (*a*_nk_*)_*k*∈*N*_ ∈ {*l*_∞_*}^*β*^ = *l*_1_* for every fixed *n* ∈ *N*, the ∗*A*-transform of *x* exists. Taking into account the hypothesis, one can easily observe that

(35) sup⁡n∈N d∗((Ax)n,θ∗)=sup⁡n∈N d∗(      ∗∑kank∗⊙xk,θ∗)≤¨||x||∞∗×¨sup⁡n∈N      β∑k||¨ank∗||¨<∞,

which leads us to the fact that *A*⊙*x* ∈ *l*_∞_*, as desired.

Conversely, suppose that *A* ∈ (*l*_∞_* : *l*_∞_*). Put *A*⊙*x* = {(*Ax*)_*n*_}_*n*∈*N*_ and observe that (*Ax*)_*n*_ is a sequence of bounded linear operators on *l*_∞_* such that sup⁡_*n*_ d*((*Ax*)_*n*_, θ*) < *∞*. Hence the results are obtained similarly from an application of Banach-Steinhaus theorem in classical mean.

**Example 21 .**

Let $(x_{k}^{*})=({\dot{\varepsilon }}_{k},{\ddot{\delta }}_{k})\in l_{\infty }^{*}$ and define the matrix *A* = (*a*_nk_*) by

(36) $$\begin{array}{c} a_{nk}^{*}∶=\{\begin{array}{cc} x_{k}^{*}, & k=n,\\ \theta^{*}, & k\neq n, \end{array} \end{array}$$

for all *k*, *n* ∈ *N*. Then $\ddot{||}a_{nk}^{*}\ddot{||}=\beta \left\{\sqrt{\varepsilon_{k}^{2}+\delta_{k}^{2}}\right\}$ holds for *k* = *n* otherwise θ*. By taking into account (*x*_k_*) ∈ *l*_∞_*, we obtain

(37) sup⁡n∈N      β∑k||¨ank∗||¨ =sup⁡n∈N{βε02+δ02,βε12+δ12,…,βεn2+δn2,…,}<∞

for all *ε*_*k*_, *δ*_*k*_ ∈ *R*. This shows, by (i) of Theorem 20, that *A* = (*a*_nk_*)∈(*l*_∞_* : *l*_∞_*).

We state and prove the Kojima-Schur theorem which gives the necessary and sufficient conditions on an infinite matrix with respect to the non-Newtonian calculus, that maps the space c* into itself. A matrix satisfying the conditions of the Kojima-Schur theorem is called a conservative matrix or convergence preserving matrix.

**Theorem 22 (Kojima-Schur).**

*A* = (*a*_nk_*)∈(c* : c*) if and only if (33) holds, and there exist *α*_*k*_, *l* ∈ C* such that

(38) $$\begin{array}{c} {\;}^{*}\underset{n\to \infty }{\lim}a_{nk}^{*}=\alpha_{k}\;for\;\;each\;\;k\in N, \end{array}$$

(39) $$\begin{array}{c} {\;}^{*}\underset{n\to \infty }{\lim}{}_{{{{\;}_{\;}}_{\;}}_{*}}\overset{}{\underset{k}{\sum }}a_{nk}^{*}=l. \end{array}$$

**Proof**

Suppose that the conditions (33), (38), and (39) hold and *x* = (*x*_k_*) ∈ c* with *x*_k_* → *s* ∈ C* as *k* → *∞*. Then, since (*a*_*nk*_)_*k*∈*N*_ ∈ {c*}^*β*^ = *l*_1_* for each *n* ∈ *N*, the ∗*A*-transform of *x* exists. In this situation, the equality

(40) $$\begin{array}{c} {}_{{{{\;}_{\;}}_{\;}}_{*}}\overset{}{\underset{k}{\sum }}a_{nk}^{*}⊙x_{k}^{*}=\left\{{}_{{{{\;}_{\;}}_{\;}}_{*}}\overset{}{\underset{k}{\sum }}a_{nk}^{*}⊙\left(x_{k}^{*}⊖s\right)\right\}⊕\left\{s⊙{}_{{{{\;}_{\;}}_{\;}}_{*}}\overset{}{\underset{k}{\sum }}a_{nk}^{*}\right\} \end{array}$$

holds for each *n* ∈ *N*. In (40), since the terms on the right hand side tend to  _∗_∑_*k*_ *α*_*k*_⊙(*x*_k_* ⊖ *s*) by (38) and the second term on the right hand side tends to *l*⊙*s* by (39) as *n* → *∞*, in the sense of ∗-limit, we have

(41) $$\begin{array}{c} {\;}^{*}\underset{n\to \infty }{\lim}{}_{{{{\;}_{\;}}_{\;}}_{*}}\overset{}{\underset{k}{\sum }}a_{nk}^{*}⊙x_{k}^{*}=\left\{{}_{{{{\;}_{\;}}_{\;}}_{*}}\overset{}{\underset{k}{\sum }}\alpha_{k}⊙\left(x_{k}^{*}⊖s\right)\right\}⊕l⊙s. \end{array}$$

Hence, *Ax* ∈ c*; that is the conditions are sufficient.

Conversely, suppose that *A* = (*a*_nk_*)∈(c* : c*). Then *A*⊙*x* exists for every *x* ∈ c*. By *e* and *e* ^(*n*)^, we denote the sequences such that *e*_*k*_ = 1* for *k* = 0,1,…, and *e*_*n*_^(*n*)^ = 1* and *e*_*k*_^(*n*)^ = θ*  (*k* ≠ *n*). The necessity of the conditions (38) and (39) is immediate by taking *x* = *e* ^(*k*)^ and *x* = *e*, respectively. Since c* ⊂ *l*_∞_*, the necessity of the condition (33) is obtained from Theorem 20(i).

**Theorem 23 .**

*A* = (*a*_nk_*)∈(*c*_0_* : c*) if and only if (33) holds and there exists (*α*_k_*) ∈ w* such that

(42) $$\begin{array}{c} {\;}^{*}\underset{n\to \infty }{\lim}d^{*}\left(a_{nk}^{*},\alpha_{k}^{*}\right)=\theta^{*} \end{array}$$

for each *k* ∈ *N*. If *A* = (*a*_nk_*)∈(*c*_0_* : c*), then (*α*_k_*) ∈ *l*_1_* and ${\;}^{*}\lim_{n\to \infty }{\;}_{*}\sum_{k}a_{nk}^{*}⊙z_{k}^{*}={\;}_{*}\sum_{k}\alpha_{k}^{*}⊙z_{k}^{*}$.

**Proof**

Suppose that (33) and (42) hold. Then there exists an *n*_*K*_ ∈ *N* for *K* ∈ *N* and $\ddot{\varepsilon }\;\;\ddot{>}\;\;0$ such that

(43) $$\begin{array}{c} {}_{{{{\;}_{\;}}_{\;}}_{\beta }}\overset{k}{\underset{k=0}{\sum }}d^{*}\left(a_{nk}^{*},\alpha_{k}^{*}\right)<\ddot{\varepsilon }, \end{array}$$

for all *n* ≥ *n*_*K*_. Since

(44)       β∑k=0kd∗(αk∗,θ∗)≤¨      β∑k=0kd∗(ank∗,αk∗)+¨      β∑k=0kd∗(ank∗,θ∗)≤ε¨ +¨ M

for *n* ≥ *n*_*K*_, by (42), one can see that (*α*_k_*) ∈ *l*_1_* and _*β*_∑_*k*_ d*(*α*_k_*, θ*) ≤ *M*_0_. Let *z* = (*z*_k_*) ∈ *c*_0_*. Then, one can choose a *k*_0_ ∈ *N* for ${\ddot{\varepsilon }}_{1}\;\;\ddot{>}\;\;0$ such that $d^{*}(z_{k}^{*},\theta^{*})\ddot{<}{\ddot{\varepsilon }}_{1}$ for each fixed *k* ≥ *k*_0_. Additionally, since $a_{nk}^{*}\overset{*}{\to }\alpha_{k}^{*}$, as *n* → *∞* by (42), we have $a_{nk}^{*}⊙z_{k}^{*}\overset{*}{\to }\alpha_{k}^{*}⊙z_{k}^{*}$, as *n* → *∞* for each fixed *k* ∈ *N*. That is to say that  *lim⁡_*n*→*∞*_ d*(*a*_nk_*⊙*z*_k_*, *α*_k_*⊙*z*_k_*) = θ*. Hence, there exists an *N* = *N*(*k*_0_) ∈ *N* such that ${\;}_{\beta }\sum_{k=0}^{k_{0}}d^{*}(a_{nk}^{*}⊙z_{k}^{*},\alpha_{k}^{*}⊙z_{k}^{*})\ddot{<}{\ddot{\varepsilon }}_{2}$ for all *n* ≥ *N*. Thus, since

(45) d∗(      ∗∑kank∗⊙zk∗,      ∗∑kαk∗⊙zk∗) ≤¨      β∑kd∗(ank∗⊙zk∗,αk∗⊙zk∗) =      β∑k=0k0d∗(ank∗⊙zk∗,αk∗⊙zk∗)  +¨      β∑k=k0+1∞d∗(ank∗zk∗,αk∗zk∗) ≤¨ε¨2+¨      β∑k=k0+1∞[d∗(ank∗⊙zk∗,θ∗)  +¨  d∗(αk∗⊙zk∗,θ∗)] ≤¨ε¨2+¨      β∑k=k0+1∞d∗(ank∗,θ∗)d∗(zk∗,θ∗)  +¨      β∑k=k0+1∞d∗(αk∗,θ∗)d∗(zk∗,θ∗) ≤¨ε¨2+¨{ε1×¨(      β∑k=k0+1∞d∗(ank∗,θ∗)+¨      β∑k=k0+1∞d∗(αk∗,θ∗))} ≤¨ε¨2+¨[ε1×¨(M+¨M0)]

for all *n* ≥ *N*, the series _∗_∑_*k*_ *a*_nk_*⊙*z*_k_* are ∗-convergent for each *n* ∈ *N* and ${}_{*}\sum_{k}a_{nk}^{*}⊙z_{k}^{*}\overset{*}{\to }{\;}_{*}\sum_{}\alpha_{k}^{*}⊙z_{k}^{*}$, as *n* → *∞*. This means that *A*⊙*z* ∈ c*.

Conversely, let *A* = (*a*_nk_*)∈(*c*_0_* : c*) and let *z* = (*z*_k_*) ∈ *c*_0_*. Then, since *A*⊙*z* ∈ c* exists and the inclusion (*c*_0_* : c*)⊂(*c*_0_* : *l*_∞_*) holds, the necessity of (33) is trivial by (iii) of Theorem 20. Now, if we take the sequence *z* ^(*n*)^ = {*z*_*k*_^(*n*)^} ∈ *c*_0_*, then *A*⊙*z* ^(*n*)^ = {*a*_nk_*}_*n*=0_^*∞*^ ∈ c* holds for each fixed *k* ∈ *N*; that is, condition (42) is also necessary. Thus, the proof is completed.

As an easy consequence of Theorem 23, we have the following corollary.

**Corollary 24 .**

*A* = (*a*_nk_*)∈(*c*_0_* : *c*_0_*) if and only if (33) holds and (42) also holds with *α*_k_* = θ* for all *k* ∈ *N*.

**Example 25 .**

Let *k*, *n*, *r* ∈ *N* and *r* ≥ 0. The Cesaro means of order r is defined by the matrix *C*_r_* = (*c*_*nk*_^∗(*r*)^) as

(46) $$\begin{array}{c} \left(c_{nk}^{*(r)}\right)=\{\begin{array}{cc} {\left(\frac{\left(\begin{array}{c} n-k+r-1\\ n-k \end{array}\right)}{\left(\begin{array}{c} n+r\\ n \end{array}\right)}\right)}^{*}; & \text{if}\;\;k\leq n\\ 0^{*}; & \text{otherwise}. \end{array} \end{array}$$

Taking *r* = 2 we obtain an infinite matrix as follows:

(47) $$\begin{array}{c} C_{2}^{*}=\left(\begin{array}{cccccc} 1^{*} & \theta^{*} & \theta^{*} & \theta^{*} & \theta^{*} & \cdots \\ {\left(\frac{2}{3}\right)}^{*} & {\left(\frac{1}{3}\right)}^{*} & \theta^{*} & \theta^{*} & \theta^{*} & \cdots \\ {\left(\frac{3}{6}\right)}^{*} & {\left(\frac{2}{6}\right)}^{*} & {\left(\frac{1}{6}\right)}^{*} & \theta^{*} & \theta^{*} & \cdots \\ \vdots & \vdots & \vdots & \vdots & \vdots & \vdots \\ {\left(\frac{2}{n+2}\right)}^{*} & {\left(\frac{2n}{\left(n+1)(n+2\right)}\right)}^{*} & \cdots & {\left(\frac{2(n-k+1)}{\left(n+1)(n+2\right)}\right)}^{*} & \theta^{*} & \cdots \\ \vdots & \vdots & \vdots & \vdots & \vdots & \vdots \end{array}\right). \end{array}$$

One can easily conclude that $\sup_{n\in N}{\;}_{\beta }\sum_{k}^{}\ddot{||}c_{nk}^{*(2)}\ddot{||}\ddot{<}\infty$ for all *k* ∈ *N* and (33) holds. On the other hand,

(48) $$\begin{array}{c} {\;}^{*}\underset{n\to \infty }{\lim}\ddot{||}c_{nk}^{*(2)}\ddot{||}={\;}^{*}\underset{n\to \infty }{\lim}\ddot{||}{\left(\frac{2(n-k+1)}{\left(n+1)(n+2\right)}\right)}^{*}\ddot{||}=\theta^{*} \end{array}$$

so (42) also holds with *α*_k_* = θ* for all *k* ∈ *N*. Therefore *C*_2_* ∈ (*c*_0_* : *c*_0_*).

A matrix satisfying the conditions of the Silverman-Toeplitz theorem is called a Toeplitz matrix or regular matrix. By (c* : c*; *p*), we denote the class of Toeplitz matrices. Now, we may give the corollaries characterizing the classes of (c* : c*; *p*).

**Corollary 26 (Silverman-Toeplitz Theorem).**

*A* = (*a*_nk_*)∈(c* : c*; *p*) if and only if (33) holds and (38) and (39) also hold with *α*_k_* = θ* for all *k* ∈ *N* and *l* = 1*, respectively.

**Example 27 .**

Example 25 can be given as an example of Silverman-Toeplitz theorem. Because the conditions (33) and (38) hold with *α*_k_* = θ*. Furthermore we have ${\;}^{*}\lim_{n\to \infty }\sum_{k}\ddot{||}c_{nk}^{*(2)}\ddot{||}=1^{*}$ and (39) also holds.

**Theorem 28 .**

*A* = (*a*_nk_*)∈(*l*_∞_* : *c*_0_*) if and only if

(49) $$\begin{array}{c} {\;}^{*}\underset{n\to \infty }{\lim}{}_{{{{\;}_{\;}}_{\;}}_{*}}\overset{}{\underset{k}{\sum }}d^{*}\left(a_{nk}^{*},\theta^{*}\right)=\theta^{*}. \end{array}$$

**Proof**

Let *A* = (*a*_nk_*)∈(*l*_∞_* : *c*_0_*) and *u* = (*u*_k_*) ∈ *l*_∞_*. Then, the series _∗_∑_*k*_ *a*_nk_*⊙*u*_k_*∗-converges to θ* for each fixed *n* ∈ *N*, since *A*⊙*u* exists. Hence, *A*_n_*∶ = {*a*_nk_*}_*k*=0_^*∞*^ ∈ {*l*_∞_*}^*β*^ for all *n* ∈ *N*. Define the sequence *u* = (*u*_k_*) ∈ *l*_∞_* by *u*_k_*∶ = (1*, 1*, 1*,…, ) for all *k* ∈ *N*. Then, *A*⊙*u* ∈ *c*_0_* which yields for all *n* ∈ *N* that

(50) ∗lim⁡n→∞      ∗∑kank∗⊙uk∗=∗lim⁡n→∞      ∗∑kank∗⊙1∗=∗lim⁡n→∞      ∗∑kank∗=θ∗.

Furthermore we obtain

(51) ∗lim⁡n→∞      ∗∑kd∗(ank∗,θ∗)=∗lim⁡n→∞      β∑k||¨ank∗||¨≤¨||¨∗lim⁡n→∞      ∗∑kank∗||¨=θ∗.

Conversely, suppose that (49) holds and *u* = (*u*_k_*) ∈ *l*_∞_*. Then, since *A*_n_* ∈ {*l*_∞_*}^*β*^ = *l*_1_* for each *n* ∈ *N*, *A*⊙*u* exists. Therefore, one can observe, by using condition (49), that

(52) ∗lim⁡n→∞d∗((Au)n,θ∗)= ∗lim⁡n→∞d∗(      ∗∑kank∗⊙uk∗,θ∗)≤¨ ∗lim⁡n→∞      ∗∑kd∗(ank⊙uk,θ∗)≤¨ ∗lim⁡n→∞      ∗∑kd∗(ank,θ∗)  ×¨  d∗(uk,θ∗)≤¨sup⁡k∈N d∗(uk,θ∗) ×¨  ∗lim⁡n→∞      ∗∑kd∗(ank,θ∗)=θ∗

which means that *A*⊙*u* ∈ *c*_0_*, as desired.

**Theorem 29 .**

*A* = (*a*_nk_*) ∈ (cs* : c*) if and only if (38) holds, and

(53) sup⁡n∈N      ∗∑kd∗(Δank∗,θ∗)<¨∞where  Δank∗=ank∗⊖an,k+1∗ ∀n,k∈N.

**Proof**

Let *x* = (*x*_k_*) ∈ cs* with _∗_∑_*k*_ *x*_k_* = *s* and *y*_k_* = _∗_∑_*j*=0_^*k*^ *x*_j_* for all *k* ∈ *N*. Define the infinite matrix *B* = (*b*_nk_*) by *B* = (*b*_nk_*) = (Δ*a*_nk_*) for all *k*, *n* ∈ *N*. Suppose that *A* ∈ (cs* : c*). Then, *A*⊙*x* exists for every *x* = (*x*_k_*) ∈ cs* and is in c*. Since this also holds for *x* = *e* ^(*k*)^ ∈ cs* for each fixed *k* ∈ *N*, the necessity of (38) is clear. Consider the following relation obtained from* m*th-partial sums of the series _∗_∑_*k*_ *a*_nk_*⊙*x*_k_* by applying Abel's partial summation. In this situation, the equalities

(54)       ∗∑k=0mank∗⊙xk∗={      ∗∑k=0m−1(Δank∗)⊙yk∗}⊕anm∗⊙ym∗={      ∗∑k=0m−1(Δank∗)⊙(yk∗⊖s)} ⊕{s⊙      ∗∑k=0m−1Δank∗}⊕anm∗⊙ym∗={      ∗∑k=0m−1(Δank∗)⊙(yk∗⊖s)} ⊕[s⊙(an0∗⊖anm∗)] ⊕anm∗⊙ym∗ ∀m,n∈N.

Therefore, we derive by passing to limit in (54) as *m* → *∞* that

(55) (Ax)n=      ∗∑kank∗⊙xk∗={      ∗∑kbnk∗⊙(yk∗⊖s)}⊕(s⊙an0∗)

for all *n* ∈ *N*. Since  *lim⁡_*n*_(*Ax*)_*n*_ exists and  *lim⁡_*n*_ *a*_*n*0_* = *α*_0_*, we see by letting *n* → *∞* in (55) that  *lim⁡_*n*__∗_∑_*k*_ *b*_nk_*⊙(*y*_k_* ⊖ *s*) also exist. This yields the fact that *B* ∈ (*c*_0_* : c*), because *y* ⊖ *s* ∈ *c*_0_* if and only if *x* ∈ cs*. Hence, the matrix *B* = (*b*_nk_*) satisfies the condition (33) which is equivalent to the condition (53); that is the condition (53) is necessary.

Conversely, suppose that conditions (38) and (53) hold. First, (53) implies *A*_n_* = (*a*_nk_*)_*k*∈*N*_ ∈ {cs*}^*β*^ = bv* ⊂ *l*_∞_* for every fixed *n* ∈ *N*; hence, *A*⊙*x* exists for every *x* ∈ cs*. Also (38) and (53) imply by Corollary 24 that *B* = (*b*_nk_*)∈(*c*_0_* : *c*_0_*). Thus, it follows from (55) that

(56) $$\begin{array}{c} {}^{*}\underset{n\to \infty }{\lim}{}_{{{{\;}_{\;}}_{\;}}_{*}}\overset{}{\underset{k}{\sum }}a_{nk}^{*}⊙x_{k}^{*}=s⊙\alpha_{0}^{*}. \end{array}$$

Hence *A* = (*a*_nk_*)∈(cs* : c*). This completes the proof.

**Theorem 30 .**

*A* = (*a*_nk_*)∈(cs* : cs*) if and only if

(57) $$\begin{array}{c} \underset{n\in N}{\sup}{}_{{{{\;}_{\;}}_{\;}}_{*}}\overset{}{\underset{k}{\sum }}d^{*}\left({}_{{{{\;}_{\;}}_{\;}}_{*}}\overset{n}{\underset{j=0}{\sum }}\Delta a_{jk}^{*},\theta^{*}\right)\ddot{<}\infty , \end{array}$$

(58) $$\begin{array}{c} {}^{*}\underset{n\to \infty }{\lim}d^{*}\left({}_{{{{\;}_{\;}}_{\;}}_{*}}\overset{}{\underset{n}{\sum }}a_{nk}^{*},\alpha_{k}^{*}\right)=\theta^{*}, \end{array}$$

where *α*_k_* ∈ C* for each *k* ∈ *N*.

**Proof**

Let *x* = (*x*_k_*) ∈ cs* and define the matrix *C* = (*c*_nk_*) by *c*_*nk*_ =  _∗_∑_*j*=0_^*n*^ *a*_jk_* as follows:

(59) $$\begin{array}{c} C=\left(\begin{array}{ccccc} a_{00}^{*} & a_{01}^{*} & a_{02}^{*} & \cdots & a_{0k}^{*}\\ a_{00}^{*}⊕a_{10}^{*} & a_{01}^{*}⊕a_{11}^{*} & a_{02}^{*}⊕a_{12}^{*} & \cdots & a_{0k}^{*}⊕a_{1k}^{*}\\ a_{00}^{*}⊕a_{10}^{*}⊕a_{20}^{*} & a_{01}^{*}⊕a_{11}^{*}⊕a_{21}^{*} & a_{02}^{*}⊕a_{12}^{*}⊕a_{22}^{*} & \cdots & a_{0k}^{*}⊕a_{1k}^{*}⊕a_{2k}^{*}\\ \vdots & \vdots & \vdots & \cdots & \vdots \\ a_{00}^{*}⊕a_{10}^{*}⊕\cdots ⊕a_{n0}^{*} & a_{01}^{*}⊕a_{11}^{*}⊕\cdots ⊕a_{n1}^{*} & a_{02}^{*}⊕a_{12}^{*}⊕\cdots ⊕a_{n2}^{*} & \cdots & a_{0k}^{*}⊕a_{1k}^{*}⊕\cdots ⊕a_{nk}^{*} \end{array}\right) \end{array}$$

for all *k*, *n* ∈ *N*. Suppose that *A* = (*a*_nk_*)∈(cs* : cs*). Then, *A*⊙*x* exists for every *x* = (*x*_k_*) ∈ cs* and is in cs*. This yields for *x* = *e* ^(*k*)^ ∈ cs* for each fixed *k* ∈ *N* that the condition (58) is necessary. It is clear that the following equality

(60) $$\begin{array}{c} {}_{{{{\;}_{\;}}_{\;}}_{*}}\overset{n}{\underset{j=0}{\sum }}{}_{{{{\;}_{\;}}_{\;}}_{*}}\overset{m}{\underset{k=0}{\sum }}a_{jk}^{*}⊙x_{k}^{*}={}_{{{{\;}_{\;}}_{\;}}_{*}}\overset{m}{\underset{k=0}{\sum }}{}_{{{{\;}_{\;}}_{\;}}_{*}}‍\overset{n}{\underset{j=0}{\sum }}a_{jk}^{*}⊙x_{k}^{*}={}_{{{{\;}_{\;}}_{\;}}_{*}}\overset{m}{\underset{k=0}{\sum }}c_{nk}^{*}⊙x_{k}^{*} \end{array}$$

derived from* n*th and* m*th-partial sums of the double series  _∗_∑_*j*__∗_∑_*k*_ *a*_jk_*⊙*x*_k_* holds for all *m*, *n* ∈ *N*. Therefore, by letting *m* → *∞* in (60) we have

(61) $$\begin{array}{c} {}_{{{{\;}_{\;}}_{\;}}_{*}}\overset{n}{\underset{j=0}{\sum }}{\left(Ax\right)}_{j}={\left(Cx\right)}_{n}\;\forall n\in N. \end{array}$$

Then, since *A*⊙*x* ∈ cs* by the hypothesis, *lim⁡_*n*_∑_*j*=0_^*n*^(*Ax*)_*j*_ exists, on the left hand side of (61), which leads us to the consequence that *C* = (*c*_nk_*)∈(cs* : c*). Therefore, condition (53) of Theorem 29 is satisfied by the matrix *C* = (*c*_nk_*) which is equivalent to condition (57).

Conversely, suppose that conditions (57) and (58) hold, which imply the existence of the *A*-transform of *x* ∈ cs*. Then, since (61) also holds, the matrix *C* satisfies the conditions of Theorem 29. Hence, *lim⁡_*n*_(*Cx*)_*n*_ exists which says by (61) that *A*⊙*x* ∈ cs*, as was desired.

**Theorem 31 .**

*A* = (*a*_nk_*)∈(c* : cs*) if and only if (58) holds and

(62) $$\begin{array}{c} \underset{n\in N}{\sup}{}_{{{{\;}_{\;}}_{\;}}_{*}}\underset{k}{\sum }d^{*}\left(\overset{n}{\underset{j=0}{\sum }}a_{jk}^{*},\theta^{*}\right)\ddot{<}\infty , \end{array}$$

(63) $$\begin{array}{c} {}_{{{{\;}_{\;}}_{\;}}_{*}}\underset{n}{\sum }{}_{{{{\;}_{\;}}_{\;}}_{*}}\underset{k}{\sum }a_{nk}^{*}\;\;\text{is}\;*\text{-}convergent,\;\forall k,n\in N_{0}. \end{array}$$

**Proof**

Let *x* = (*x*_k_*) ∈ c* and define the matrix *C* = (*c*_nk_*) as in the proof of Theorem 30. Suppose that *A* = (*a*_nk_*)∈(c* : cs*). Then, *A*⊙*x* exists for every *x* ∈ c* and is in cs*. This yields for *x* = *e* ^(*k*)^ ∈ c* and *x* = *e* ∈ c* which give the necessity of the conditions (58) and (63), respectively. It is clear that we have the relation (61), derived by the same way used in the proof of Theorem 30. Then, since *A*⊙*x* ∈ cs*, that is, the series _∗_∑_*j*_(*Ax*)_*j*_  ∗-converges by the hypothesis  *lim⁡_*n*__∗_∑_*j*=0_^*n*^(*Ax*)_*j*_ exists, on the left hand side of (61), which leads us to the consequence that *C* = (*c*_nk_*)∈(c* : c*). Therefore, condition (33) in Kojima-Schur theorem, is satisfied by the matrix *C* = (*c*_nk_*) which is equivalent to condition (62).

Conversely, suppose that conditions (58), (62), and (63) hold, which imply the existence of the *A*-transform of *x* ∈ c*. Then, since (61) also holds, the matrix *C* = (*c*_nk_*) satisfies the conditions of Kojima-Schur theorem. Hence, *lim⁡_*n*_(*Cx*)_*n*_ exists which says by (61) that *A*⊙*x* ∈ cs*, as was desired.

## 4. Conclusion

At the beginning of 1981, the wage-rate in dollars per hour at a certain company was *w*_0_ and the cost of living index for the United States was *c*_0_. At the end of 1981, the amounts were *w*_1_ and *c*_1_, respectively. Company and union negotiators had agreed at the beginning of 1981 that, thereafter, the wage-rate would be adjusted to reflect changes in the cost of living index. Assuming that the cost of living index is always increasing and that the wage-rate changes “uniformly” and continuously relative to the cost of living index, find the wage-rate *w*_*t*_ at time *t* in terms of the constants *c*_0_, *c*_1_, *w*_0_, and *w*_1_ the cost of living index *c*_*t*_ at time *t*. There is no unique solution to this problem; we shall give two reasonable solutions (cf. [14]).

Firstly, since the wage-rate changes “uniformly” relative to the cost of living index, we may reasonably assume that equal differences in the cost of living index give rise to equal differences in the wage-rate. Furthermore, since the changes are “continuously” relative to the cost of living index, it can be proved that

(64) $$\begin{array}{c} w_{t}=w_{0}+\left[\frac{w_{1}-w_{0}}{c_{1}-c_{0}}\right]\left(c_{t}-c_{0}\right). \end{array}$$

Secondly, since the changes are “continuously” relative to the cost of living index, it can be given that

(65) $$\begin{array}{c} w_{t}=w_{0}{\left[{\left(\frac{w_{1}}{w_{0}}\right)}^{1/\left(\ln c_{1}-\ln c_{0}\right)}\right]}^{\ln c_{t}-\ln c_{0}}. \end{array}$$

We shall see that the expression within the brackets represents a new gradient that plays a fundamental role in the bigeometric calculus which is a branch of non-Newtonian calculus. On the other hand, in the mathematical solution of many fundamental physical problems we are naturally led to series whose terms contain factors which are the mathematical representations of the damping factors of the physicist. These same factors may be interpreted as convergence factors for summable series, since they satisfy the conditions of the general theorems. Thus, the use of convergence factor theorems and the theory of summable series frequently serves to extend the domain of applicability of the mathematical solution of physical problems.

The table on the characterizations of the matrix transformations between certain spaces of sequences with real or complex terms was given by Stieglitz and Tietz [26]. To prepare the corresponding table for certain sequence spaces over the non-Newtonian complex field C*, we characterize some classes of infinite matrices. Of course, to complete the table of matrix transformations from the set *μ*_1_* to the set *μ*_2_*, there are several open problems depending on the choice of generator functions.

The main results given in final section of the present paper will be based on examining the domain of some matrices in the classical sets of sequences. This is a new development of the matrix transformations between sequence spaces over C*. Finally, we should note from now on that our next papers will be devoted to the matrix domains of the classical sets of sequences over C*.
